# Supplementary material for: Mimicking kidney flow shear efficiently induces aggregation of LECT2, a protein involved in renal amyloidosis
Source: J Biol Chem. 2024 Mar 26;300(5):107231. doi: 10.1016/j.jbc.2024.107231 (PMC11040205; doi:10.1016/j.jbc.2024.107231)
Supplement: Supplemental Figures S1–S14 [file mmc1.pdf]

Supporting Information for:

Mimicking Kidney Flow Shear Efficiently Induces Aggregation of LECT2, a Protein Involved in Renal Amyloidosis

Jeung-Hoi Ha[#1], Yikang Xu[#2], Harsimranjit Sekhon[1], Wenhan Zhao[2], Stephan Wilkens[1], Dacheng Ren[*2,3,4], and Stewart N. Loh[*1]

[1]Department of Biochemistry and Molecular Biology, State University of New York Upstate Medical University, Syracuse, NY 13210

[2]Department of Biomedical and Chemical Engineering, Syracuse University, Syracuse, NY 13244

[3]Department of Civil and Environmental Engineering, Syracuse University, Syracuse, NY 13244

[4]Department of Biology, Syracuse University, Syracuse, NY 13244

Contents:

Supporting Figures S1 – S14, related to Figure 2, Figure 3, Figure 7, and Table 1.

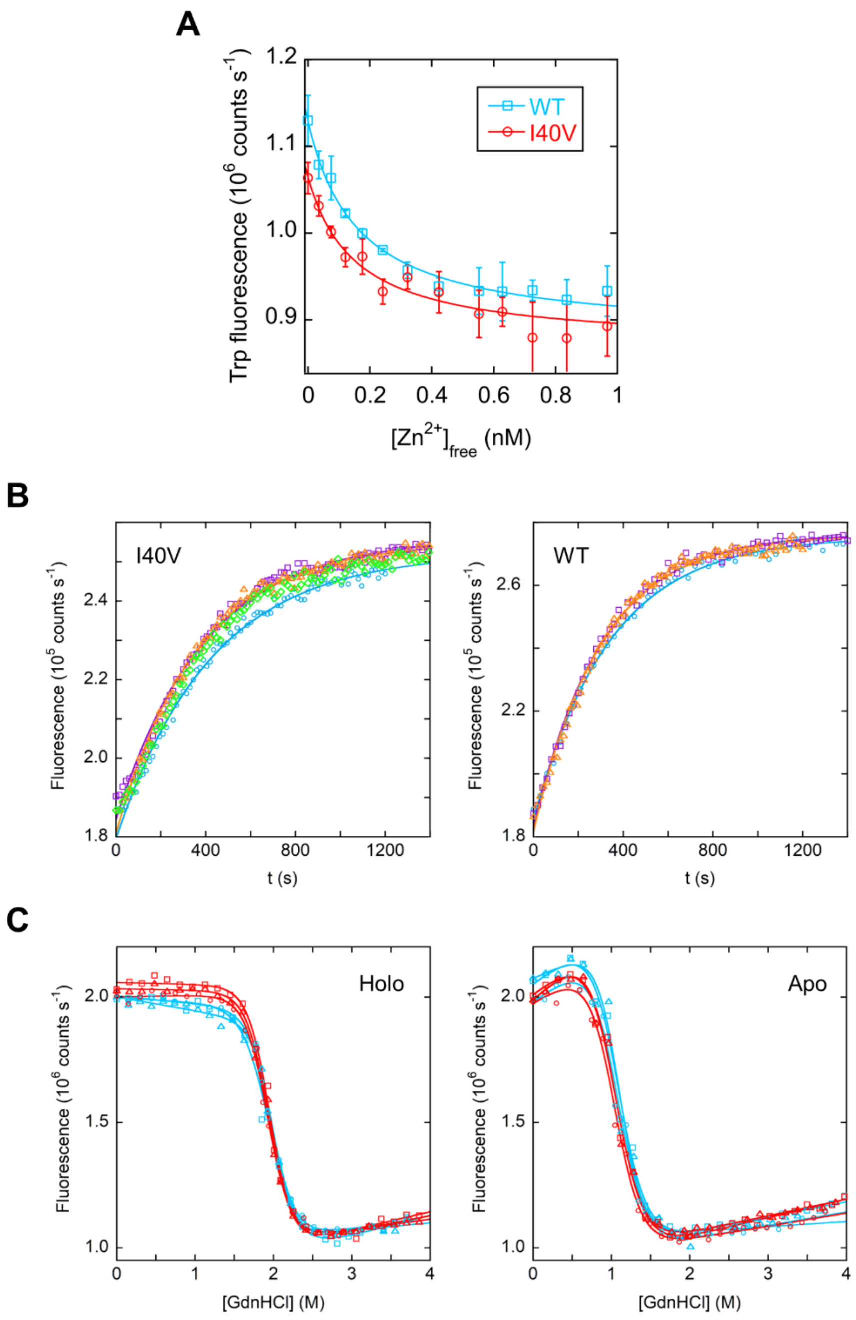

Supporting Figure S1, related to Table 1. Zinc binding and stability of LECT2. (A) I40V and WT LECT2 bound zinc with comparable affinities. Lines are best fits to the quadratic binding equation. Error bars are s.d. (n = 3). **(B)** I40V and WT LECT2 exhibited similar off-rates. Lines are best fits to a single exponential function. **(C)** The I40V mutation selectively destabilized the apo form of LECT2 compared to the holo form. Red and blue data indicate I40V and WT proteins, respectively. Lines are best fits to the linear extrapolation equation for two-state denaturation.

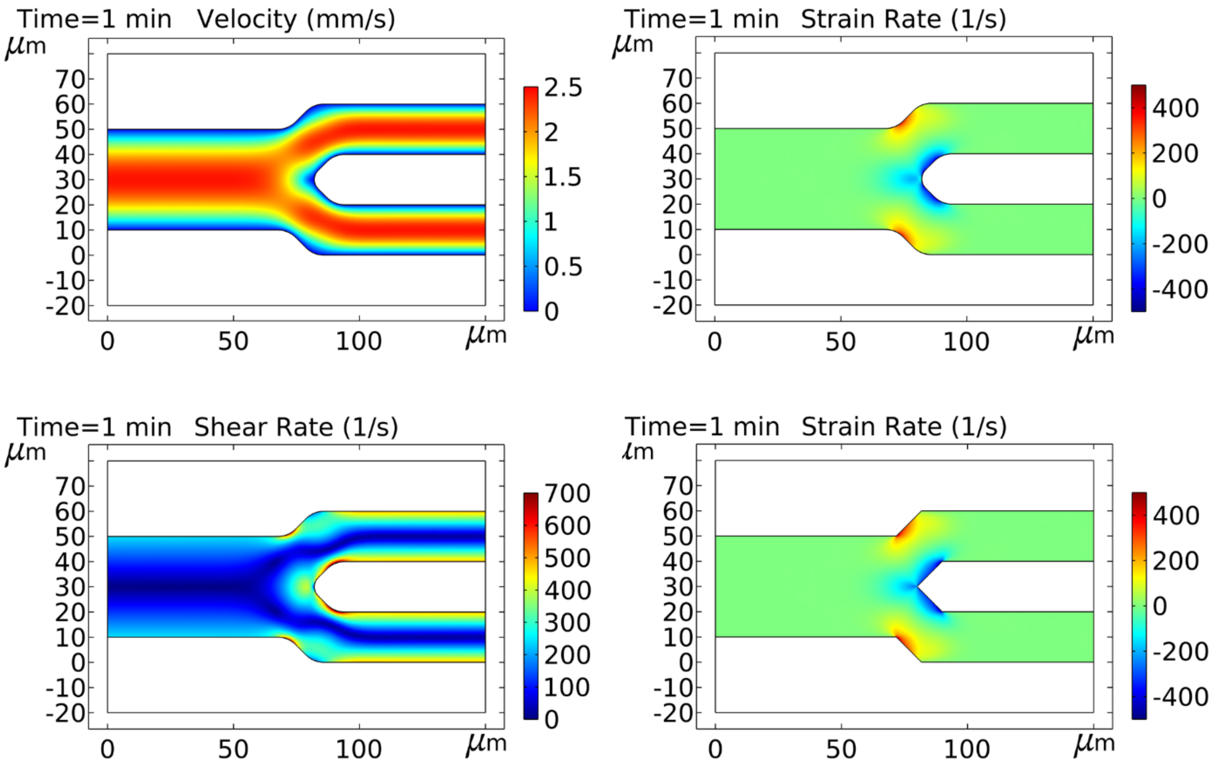

Supporting Figure S2, related to Figure 1. Simulations of flow shear and flow strain in devices with rounded and angular channels. The same 40/20 µm junctions as depicted in the right panels of Figure 1C and Figure 1D are shown, except here with rounded junctions (the angular channel device is shown at lower right for comparison). COMSOL was used to calculate velocities, shear rates, and strain rates.

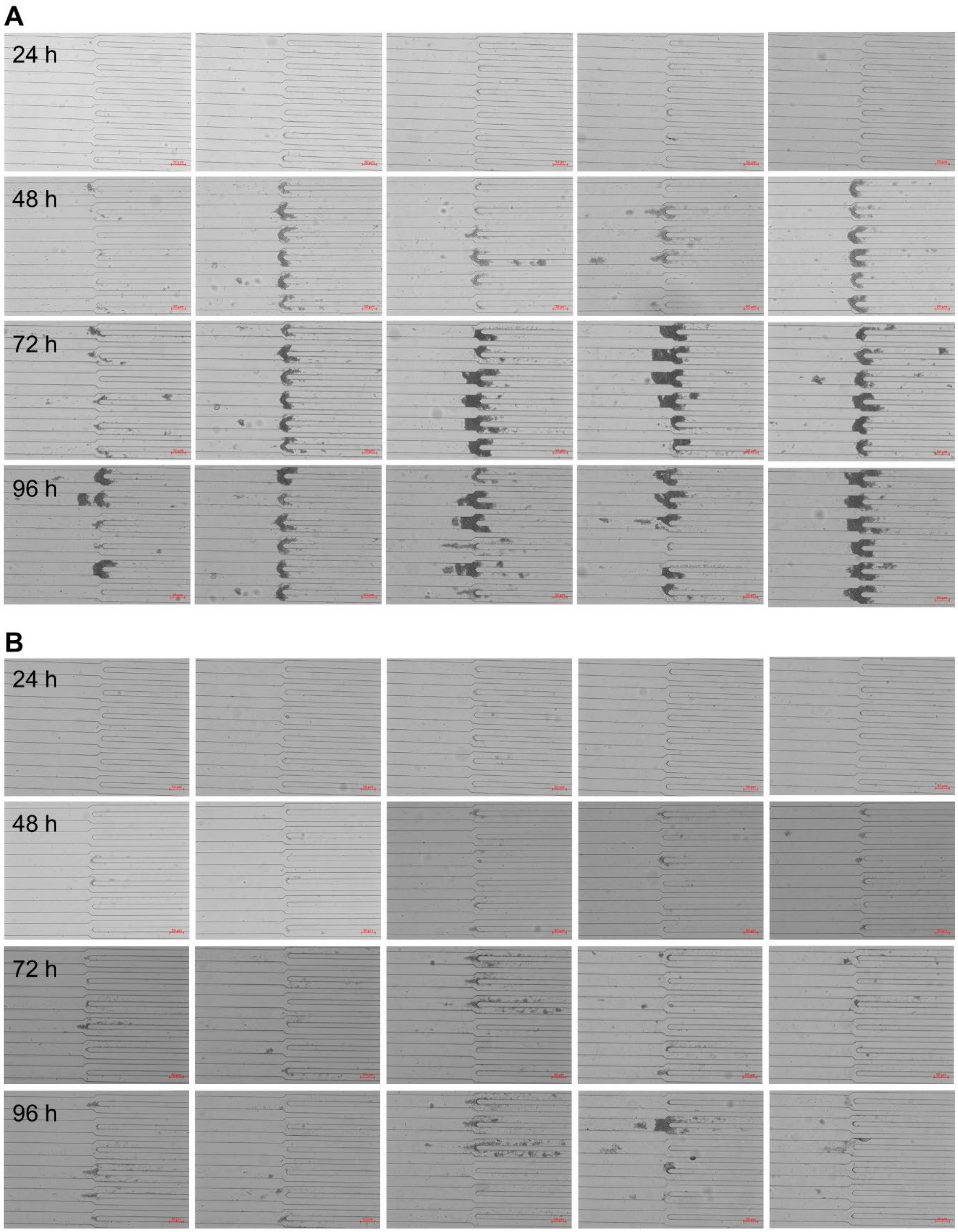

Supporting Figure S3, related to Figure 2. Images of 40/20 µm junctions from the microfluidic chip, obtained from a second BR. I40V and WT apoLECT2 are shown in **(A)** and **(B)**, respectively. Scale is provided by the red bars (50 µm) and by the widths of the channels (40 µm and 20 µm). See Experimental Procedures for description of BR.

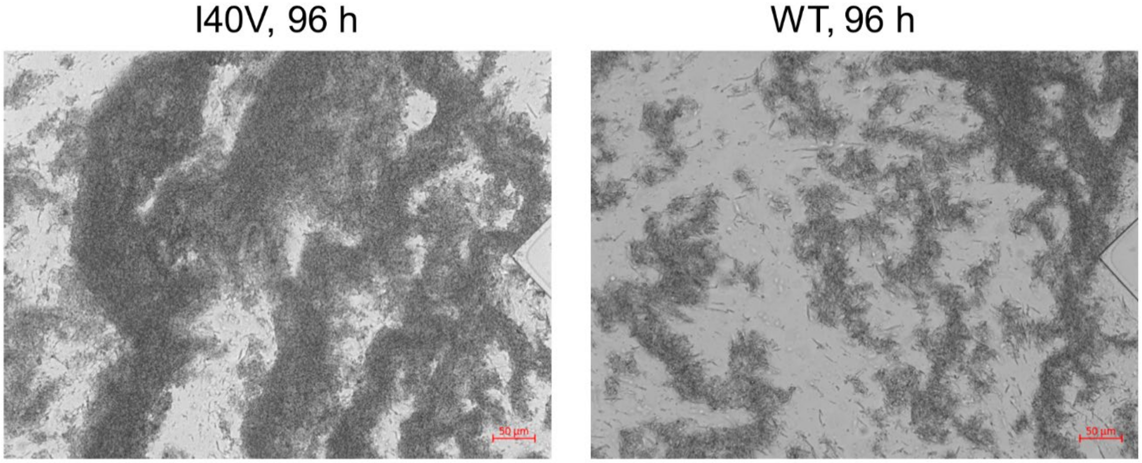

Supporting Figure S4, related to Figure 2. Images of 1280/640 µm junctions from the microfluidic chip. Scale bars are 50 µm.

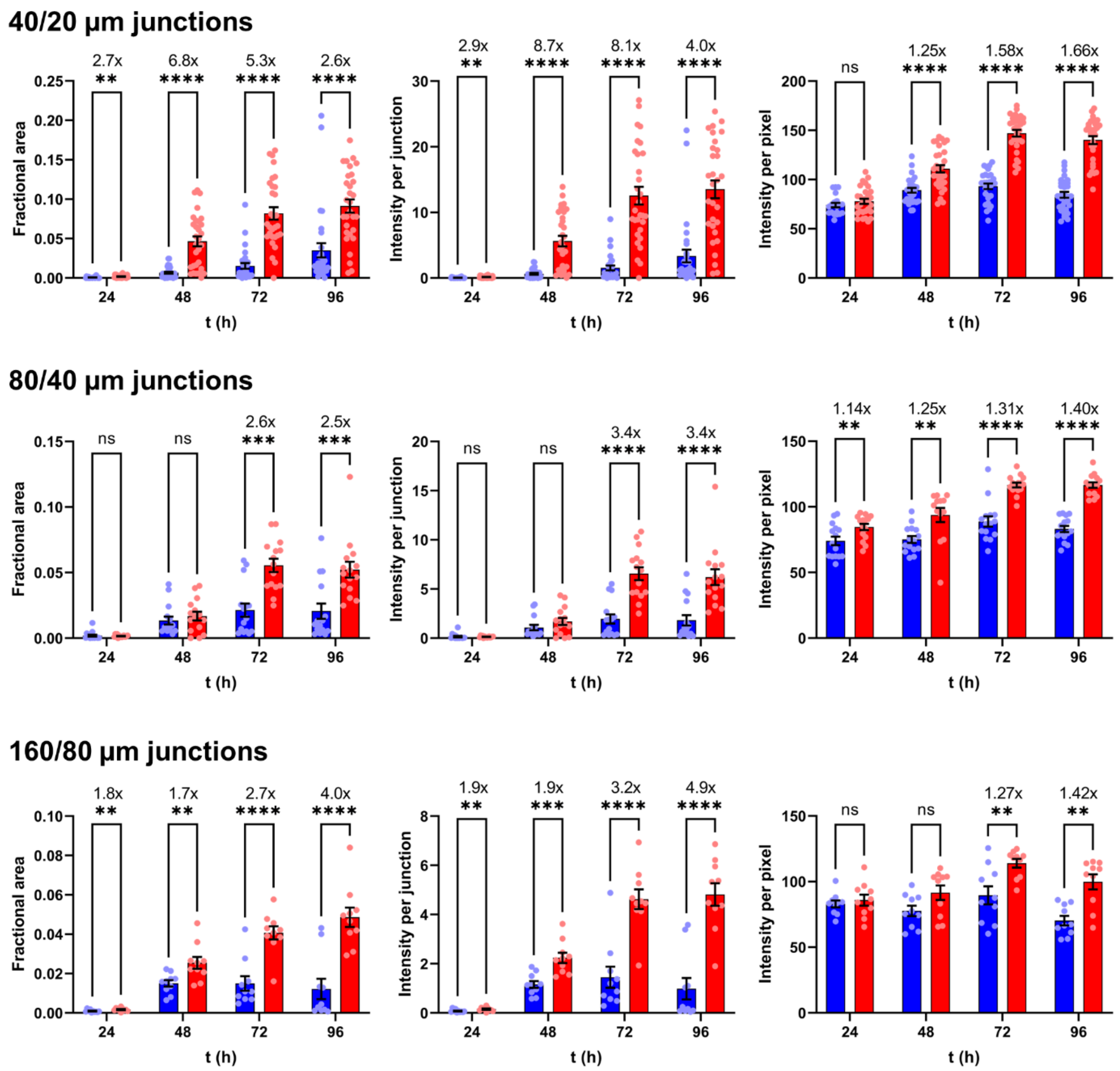

Supporting Figure S5, related to Figure 3. Quantification of microfluidic aggregates from an additional BR. Red and blue data indicate I40V and WT apoLECT2, respectively. See Figure 3 for description of statistical analysis. See Experimental Procedures for description of BR.

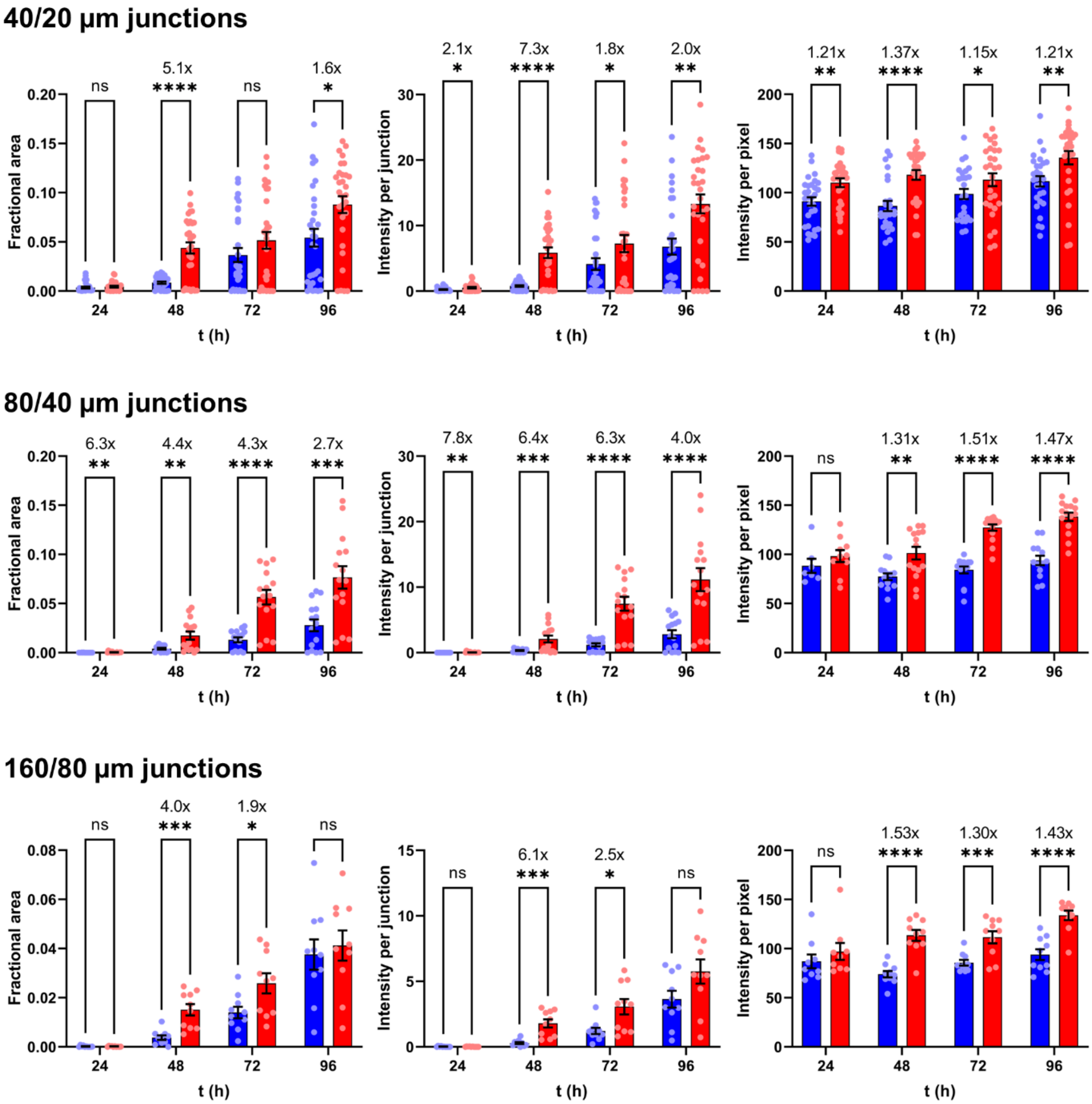

Supporting Figure S6, related to Figure 3. Quantification of microfluidic aggregates from an additional BR. Red and blue data indicate I40V and WT apoLECT2, respectively. See Figure 3 for description of statistical analysis. See Experimental Procedures for description of BR.

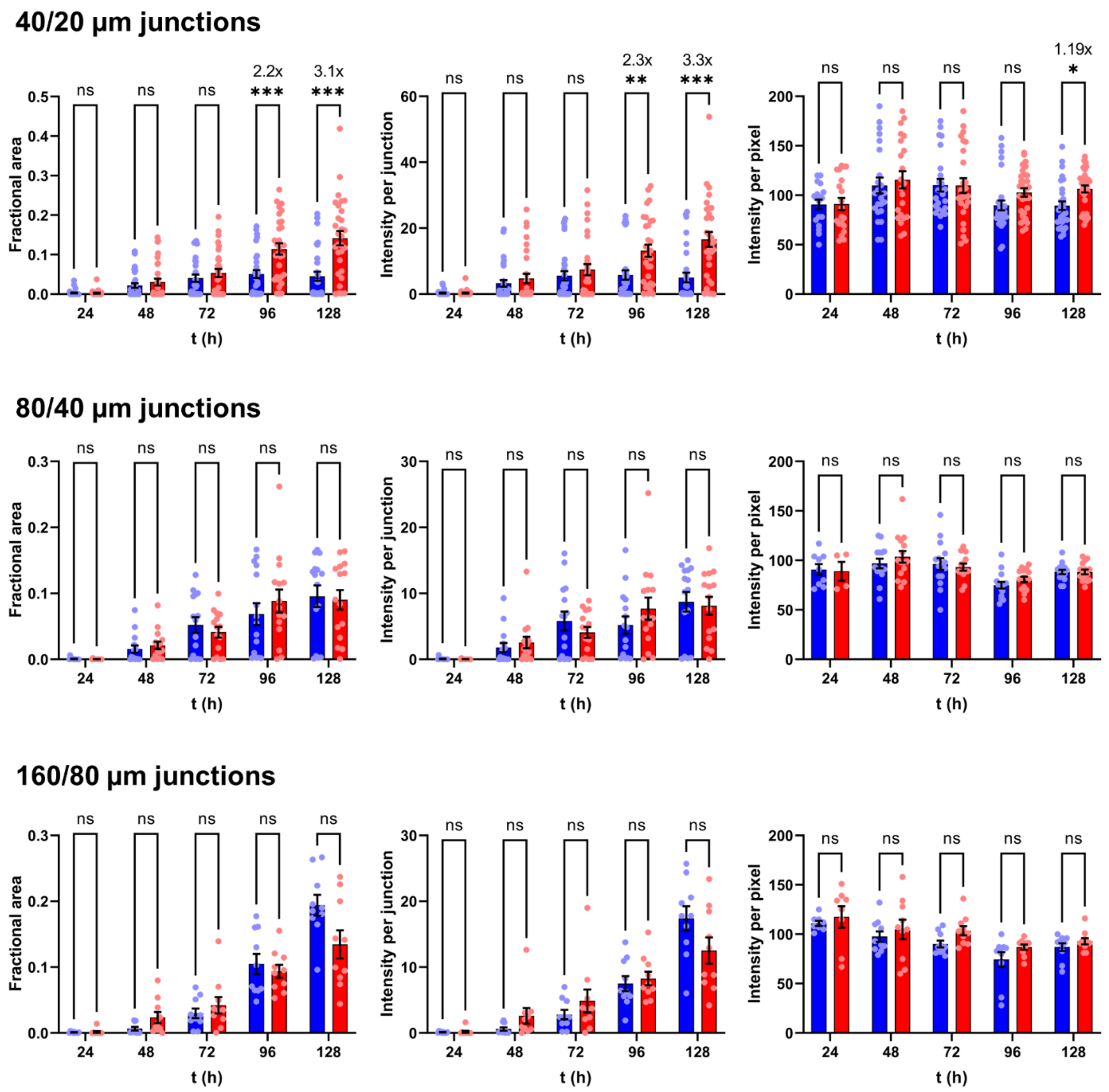

Supporting Figure S7, related to Figure 3. Quantification of microfluidic aggregates from an additional BR. Red and blue data indicate I40V and WT apoLECT2, respectively. See Figure 3 for description of statistical analysis. See Experimental Procedures for description of BR.

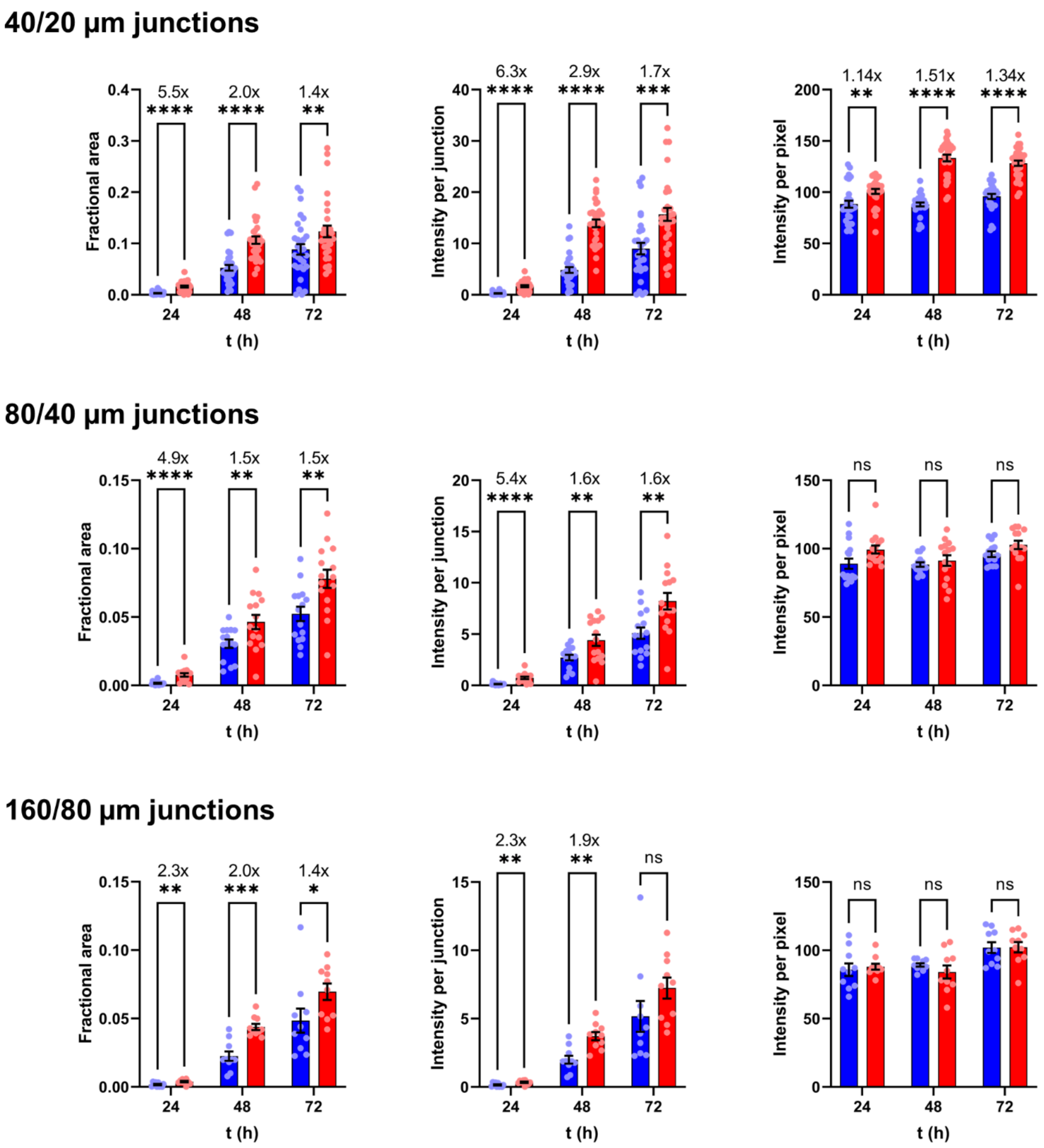

Supporting Figure S8, related to Figure 3. Quantification of microfluidic aggregates from an additional BR. Red and blue data indicate I40V and WT apoLECT2, respectively. See Figure 3 for description of statistical analysis. See Experimental Procedures for description of BR.

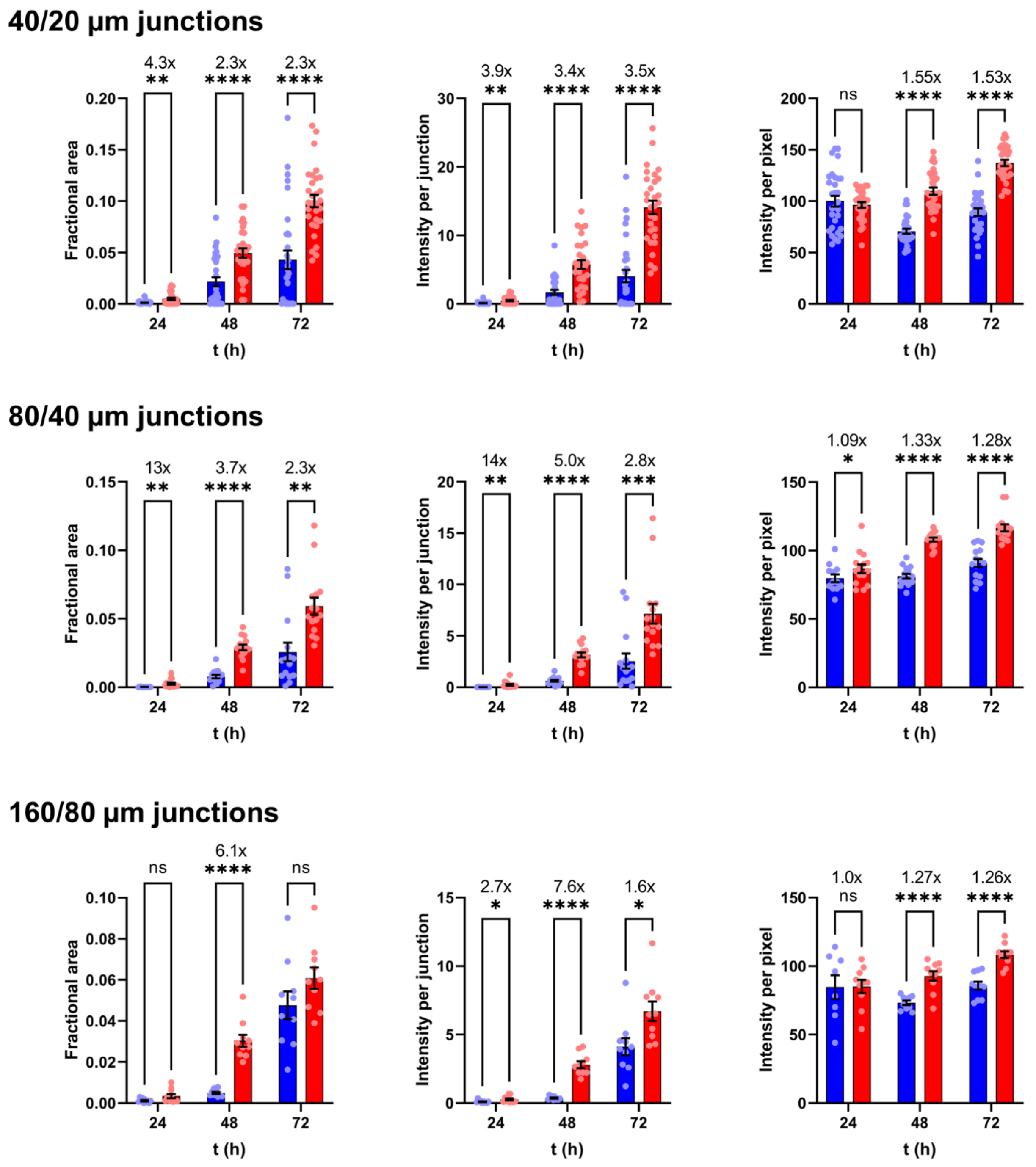

Supporting Figure S9, related to Figure 3. Quantification of microfluidic aggregates from an additional BR. Red and blue data indicate I40V and WT apoLECT2, respectively. See Figure 3 for description of statistical analysis. See Experimental Procedures for description of BR.

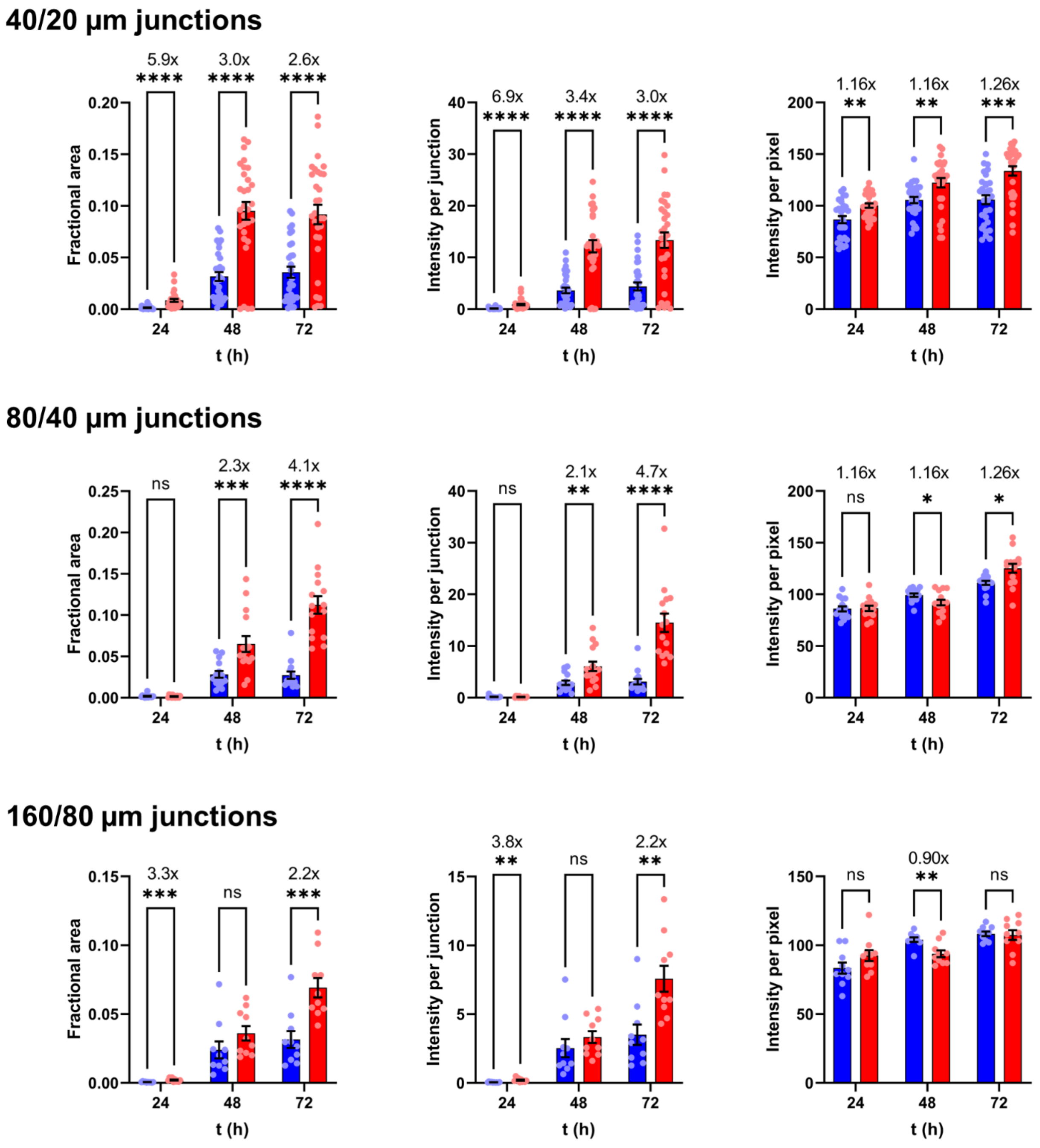

Supporting Figure S10, related to Figure 3. Quantification of microfluidic aggregates from an additional BR. Red and blue data indicate I40V and WT apoLECT2, respectively. See Figure 3 for description of statistical analysis. See Experimental Procedures for description of BR.

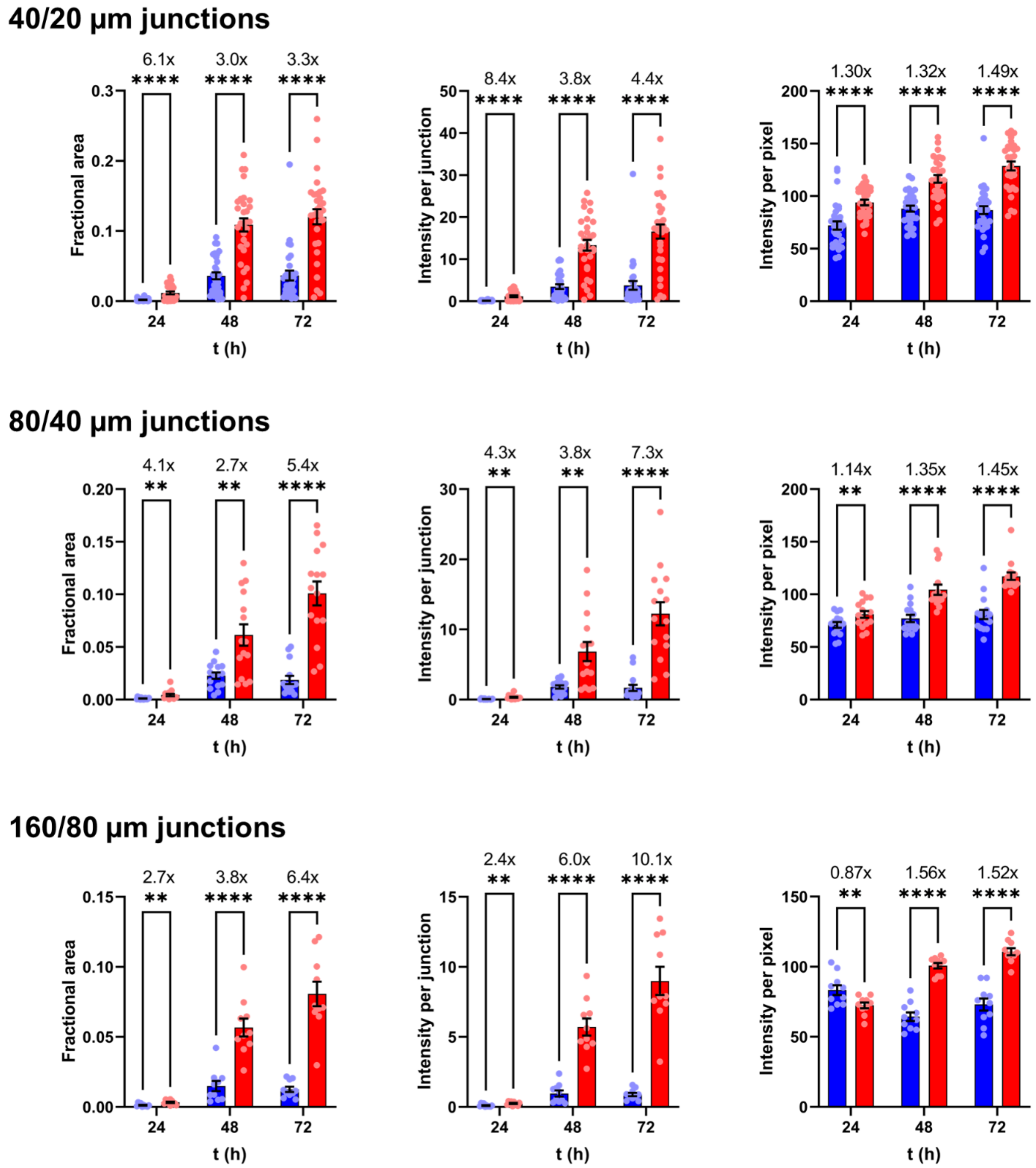

Supporting Figure S11, related to Figure 3. Quantification of microfluidic aggregates from an additional BR. Red and blue data indicate I40V and WT apoLECT2, respectively. See Figure 3 for description of statistical analysis. See Experimental Procedures for description of BR.

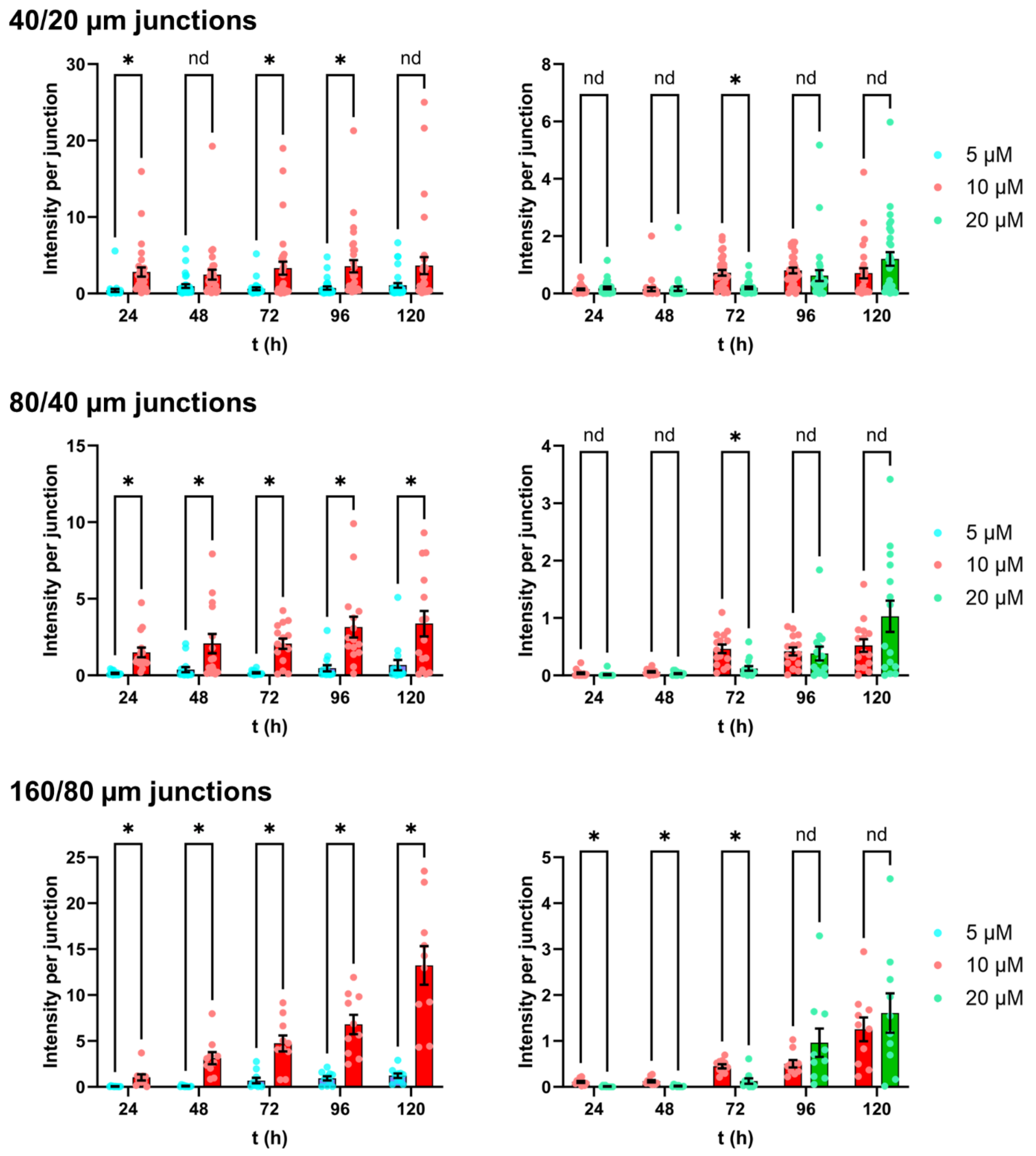

Supporting Figure S12, related to Figure 3. Dependence of microfluidic aggregation on protein concentration. Experiments were performed and data were analyzed as in Figure 3, at the I40V apoLECT2 concentrations indicated at right.

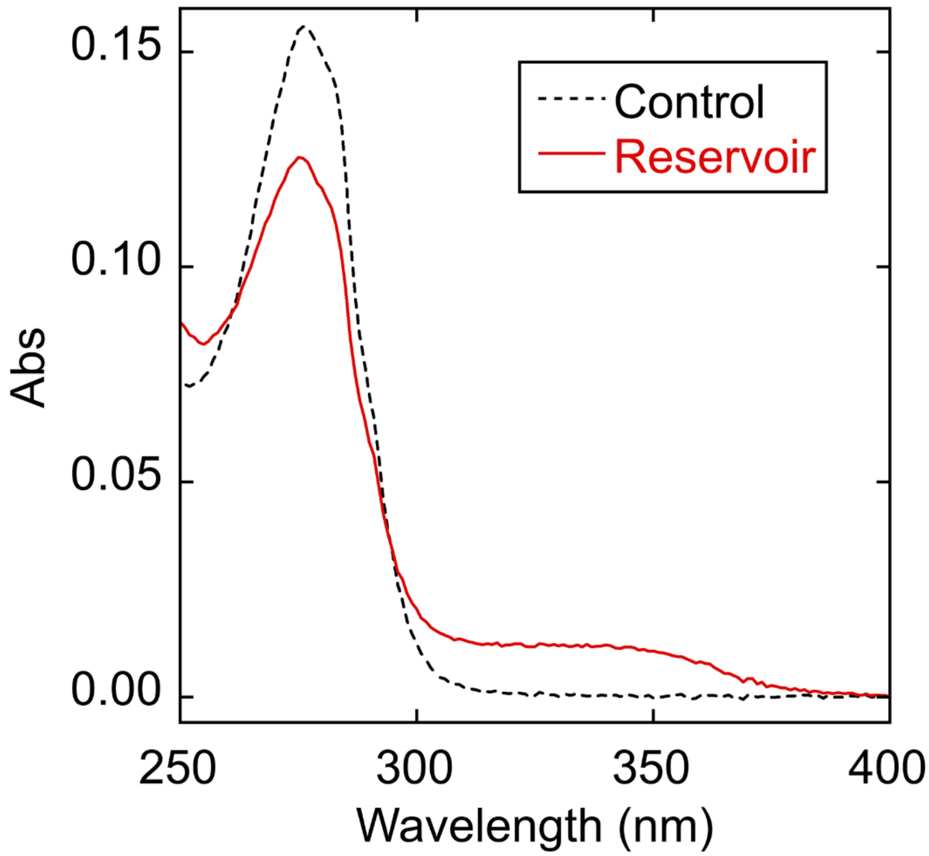

Supporting Figure S13, related to Figure 2 and Figure 7. Absorbance scans of reservoirs from I40V apoLECT2 microfluidic experiments shown in Figure 2. The control spectrum is that of an identical sample that had not been passed through the device.

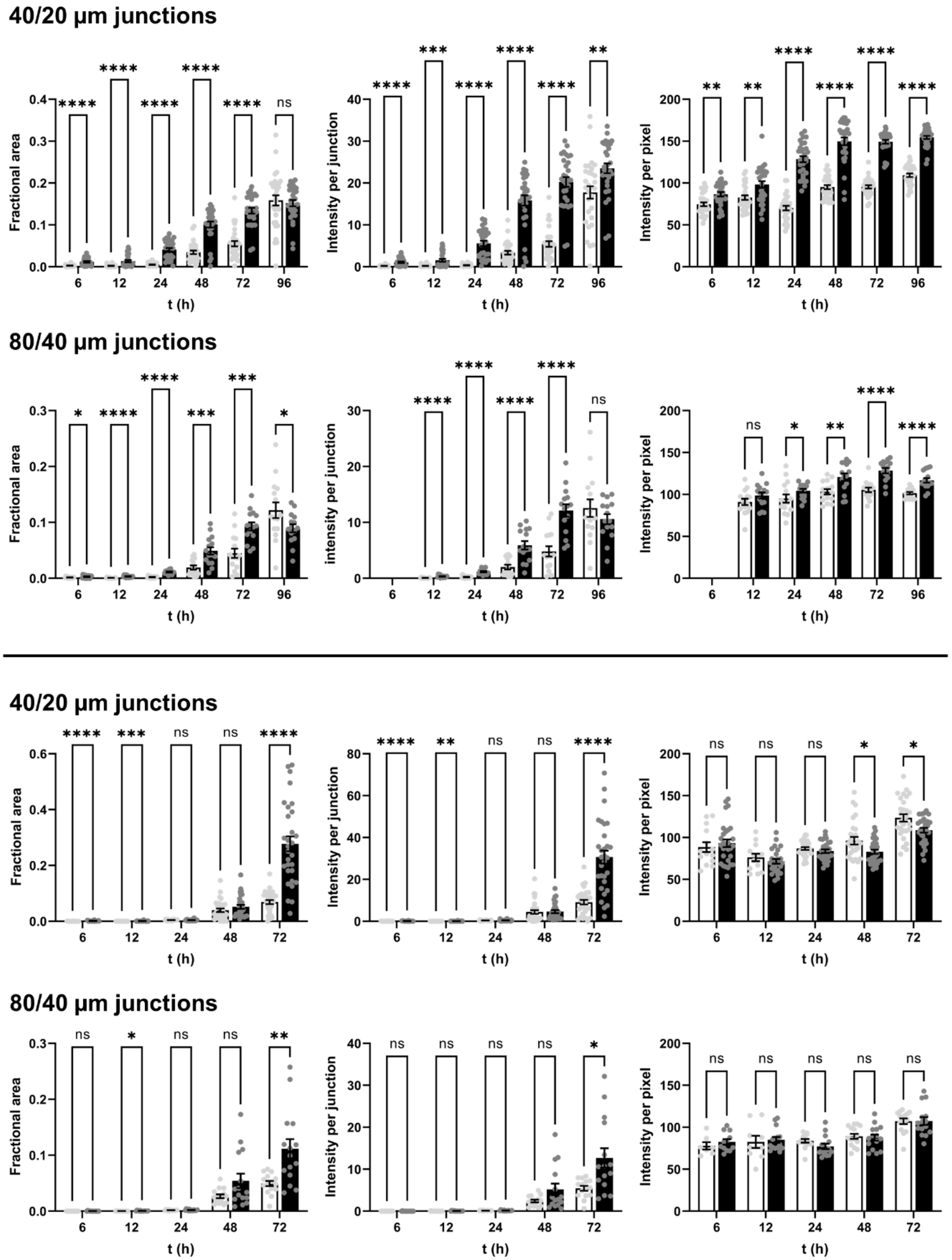

Supporting Figure S14, related to Figure 7. Quantification of microfluidic aggregates from two additional BRs. Black and white bars indicate seeded and unseeded (respectively) samples of I40V apoLECT2. See Figure 6 for description of statistical analysis.
